# Adhesion-Related Phenomena of Stellite 6 HVOF Sprayed Coating Deposited on Laser-Textured Substrates

**DOI:** 10.3390/ma17205069

**Published:** 2024-10-17

**Authors:** Žaneta Dlouhá, Josef Duliškovič, Marie Frank Netrvalová, Jana Naďová, Marek Vostřák, Sebastian Kraft, Udo Löschner, Jiří Martan, Šárka Houdková

**Affiliations:** 1 Research and Testing Institute Plzen, Tylova 1581/46, 301 00 Pilsen, Czech Republic; dlouha@vzuplzen.cz (Ž.D.); duliskovic@vzuplzen.cz (J.D.); netrvalova@vzuplzen.cz (M.F.N.); nadova@vzuplzen.cz (J.N.); vostrak@vzuplzen.cz (M.V.); 2 Laserinstitut Hochschule Mittweida, University of Applied Sciences Mittweida, Schillerstraße 10, 09648 Mittweida, Germanyloeschne@hs-mittweida.de (U.L.); 3 New Technologies Research Centre (NTC), University of West Bohemia, Teslova 1198/9, 301 00 Pilsen, Czech Republic; jmartan@ntc.zcu.cz

**Keywords:** thermal spray, adhesion, laser texturing, HVOF, grit blasting, carbon steel, nitrided steel

## Abstract

The focus of this research is to examine the feasibility of using laser texturing as a method for surface preparation prior to thermal spraying. The experimental part includes the thermal spraying of a Stellite 6 coating by High Velocity Oxygen Fuel (HVOF) technology on laser-textured substrates. The thermal spraying of this coating was deposited both on conventional substrate material (low carbon steel) and on substrates that had been previously heat treated (nitrided steel). The properties of the coatings were analysed using scanning electron microscopy (SEM), optical microscopy (OM) and Raman spectroscopy. Adhesion was assessed through a tensile adhesion test. The results showed the usability of laser texturing in the case of carbon steel, which was comparable or even better than traditional grit blasting. For nitrided steel, the problem remains with the hardness and brittleness of the nitrided layer, which allows for the propagation of brittle cracks near the interface and thus reduces the adhesion strength.

## 1. Introduction

Thermal spraying is a field of surface treatment that uses various technologies to create functional coatings on the surface of different types of components. The principle of the thermal spraying process is to form a coating by melting the material in the form of a powder, wire or suspension. This molten material is then accelerated towards the coated surface. The impact of the material particles on the surface produces a coating with a specific structure and desired functional properties. The coating thus formed can be deposited on a wide range of different materials such as metals, ceramics or polymers. The properties of the coatings depend on the deposited material, the particle size, the temperature of the particles and the velocity of the particles prior to the impact on the substrate surface [1]. In order to maximize the lifetime of a thermal sprayed component, the mechanical and physical–chemical adhesion forces must be high [2]. Appropriate surface treatment of the substrate is required for proper attachment of the coating particles. Conventional surface preparation methods consist of cleaning the surface to remove contaminants and roughening the surface for better adhesion.

High Velocity Oxygen Fuel (HVOF) technology uses a special torch in which the exhaust gas expands rapidly at the end of the nozzle, thus dramatically accelerating it. With the appropriate choice of powder feed parameters and ensuring its transport to the centre of the flame, the particles are accelerated to supersonic speeds [3]. Coatings deposited by HVOF technology are characterized by high adhesion and density, low oxide and pore content and high cohesive strength. The advantages of HVOF deposited coatings are mainly due to the low porosity, residual compressive stresses in the surface layer of the coating and high adhesion to the substrate material [4,5]. The adhesion of thermal sprayed coatings strongly depends on the substrate topography, substrate temperature and surface composition. Also, surface cleanliness is a key parameter for the quality of the deposited coating [6,7]. In general, oxides, carbon and oils must be removed from the surface as they alter the physical–chemical properties [8]. An example of such a standard preparation procedure before thermal spraying may be grit blasting. This process involves the impact of abrasive particles (for example Al_2_O_3_) on the surface of the substrate [9]. The processing rate of this treatment typically ranges from 400 to 1000 mm^2^/s. From an economic point of view, one of the disadvantages of this procedure is the high consumption of abrasive material [10]. Another disadvantage of this processing method is the risk of corundum particles being trapped in the substrate, where the particles act as stress concentrators [11,12]. For ductile substrates, surface or subsurface embrittlement can occur. These entrapped particles also adversely affect the resulting adhesion of the coating. In view of the growing environmental concerns of recent years, the elimination of chemical waste from cleaning is another disadvantage of the grit blasting process [13,14].

Laser texturing technology, which improves the adhesion of the coating by mechanical and chemical bonding, appears to be a promising alternative to standard surface preparation before the thermal spraying [15,16,17]. This technology consists of focussing a laser beam into a small spot [13]. In this way, micro- or nanostructures of different geometries can be formed on the surface of the substrate. Laser treated surfaces have a high level of roughness and cleanliness [10]. Advantages of laser texturing technology include the absence of inclusions and particles, a limited affected zone, high quality interface, high flexibility, precision, surface modification at very small thicknesses, a large number of techniques (texturing, roughening, cleaning, etc.), low environmental impact and no chemical waste [2,11]. The other advantages of lasers are the ability to perform very localized treatments and the ease of application in an industrial process [18]. In addition, this contactless process can be applied to the “hard to reach” areas [19]. Laser surface treatments can be applied before, during or after the thermal spraying, resulting in a wide range of coating property improvements in microstructure, adhesion strength, etc.

Laser pre-treatment technologies have a positive effect on coating adhesion. Feng et al. [20] investigated the effect of laser texturing on the properties of 30CrMnSiA. The results of their study showed that regularly repeated texture increased both roughness and tensile strength. Lamraoui et al. [21] used a pulsed Nd:YAG laser to texture aluminum substrate Al 2017. According to their results, texture parameters (diameter and depth) have a strong influence on coating adhesion. Zhan et al. [22] created different textures using an IPG laser at 400 mm/s speed. In the case of sinusoidal texture, a higher adhesion of plasma-sprayed coating was achieved than that of the grit-blasted sample. The laser treatment carried out during thermal spraying causes modification of the microstructure of the coating and thus allows for improved mechanical properties and increased wear and corrosion resistance. Laser treatments carried out after thermal spraying can improve the density and adhesion strength of the coatings. They can also cause phase transformation and refinement of the structure [13].

The adhesion of thermal spray coatings on laser-textured substrates is highly influenced by the texture geometry. In particular, the optimum texturing dimensions (pitch, diameter and depth) must be adapted to the average size of the deposited powder [13]. Through laser-textured surfaces with optimized texture morphology, higher adhesion strength values can be achieved than those generally observed after conventional surface treatment [8].

In this paper, two different surface treatments were used before the thermal spraying—laser texturing and grit blasting. Grit blasting is the most commonly used method of surface preparation prior to the application of thermal spraying. By selecting appropriate parameters such as material type, abrasive grain size, distance, angle and pressure, the resulting surface structures can be modified [23]. For economic and ecological reasons, there are increasing efforts to replace this method with another technology. Grit blasting was added for comparison with the innovative laser-texturing technique. It was also tested whether it is possible to grit blast a nitrided substrate and whether there is any effect on the resulting adhesion of the coating.

The materials chosen for this experiment were low carbon steel as one of the most commonly used substrates and Co-based alloy Stellite 6^®^ (Höganäs, Halmstad, Sweden) as the coating. Stellite 6^®^ is a widely used material in the energy and engineering industries. In addition, nitrided steel was used as a substrate to verify the applicability of laser texturing to ensure the adhesion of the coating to the hardened substrate material. Such a possibility would increase the applicability of thermally sprayed (TS) coatings, namely for the restoration of worn hardened surfaces.

## 2. Materials and Methods

Low carbon steel (1.0570) and low-alloy noble chrome-molybdenum steel for refining steel (42CrMo4) substrates were selected for this experiment. Table 1 contains the chemical composition of both substrates. Table 2 contains the mechanical properties of both substrates. The samples used were circular in shape with a diameter of 25 mm and a thickness of 7.5 mm.

The (42CrMo4) steel substrate samples were subjected to nitriding, which is described in detail in Section 2.1.

The hardness of the substrate surface was 64.7 ± 1.3 HV and 87.2 ± 0.8 HV for low carbon steel and nitride 42CrMo4 steel, respectively.

The surface of both substrate materials was prepared for coating deposition by means of grit blasting and laser texturing. The details of the substrate preparation procedure are given in Section 2.2.

Stellite 6 (Amperit 2637-02) coating was deposited on both above-mentioned substrates. The Stellite 6 powder used was from Höganäs (Höganäs, Halmstad, Sweden). The particle size of the powder ranged from 20 to 53 µm. Stellite 6 is a cobalt-based alloy that consists of carbide phases in a CoCr matrix. This material is characterized by its resistance to wear and scratch formation and excellent corrosion resistance. It is mainly used as protection against mechanical and chemical damage over a wide temperature range. The process of coating deposition is described in detail in Section 2.3.

### 2.1. Parameters of Nitriding Process

Part of the experiment was thermal spraying on a pre-nitrided substrate made of 42CrMo4 steel. The nitriding process of the samples was carried out in a standard electric heat treatment furnace at a temperature of 515 °C, holding at 30 h with 30% dissociation.

### 2.2. Substrate Preparation

Both substrates were subjected to the treatment of the substrate to enhance the adhesion of the Stellite 6 coating. The grit blasting was performed in VZU Plzen, according to a standardized procedure. A total of three sets of grit-blasted samples were added for comparison (two nitrided sets and one without the nitriding). The corundum of size F22 and pressure 4–6 bar was used, to reach the surface roughness of 10.4 ± 0.4 Ra and 7.4 ± 0.4 Ra on the carbon steel and nitride steel, respectively. The overview of grit-blasted samples is summarized in Table 3.

**Figure 1:**
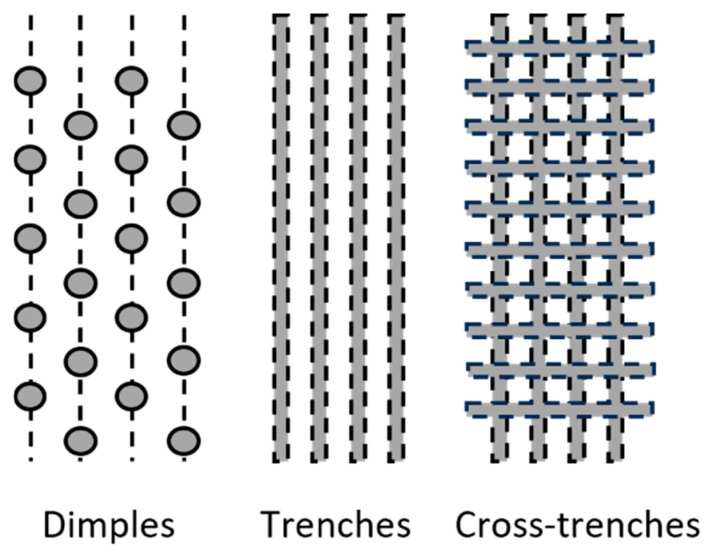
Laser texturing patterns.

The process of laser texturing of the samples was performed in Laserinstitut Hochschule Mittweida. The nominal parameters of the lasers used are shown in Table 4. Structures of different geometries were textured on the samples. Figure 1 shows the geometry of the laser textures used. All geometries were created to match the parameters of the grit-blasted surfaces. Dimple geometries were produced using a 200 W/ns laser. For pulsed lasers, the scanning speed depends on the laser source and geometry of the element. Dimples are created by repeated laser irradiation of the same spot on the surface [23].

**Figure 2:**
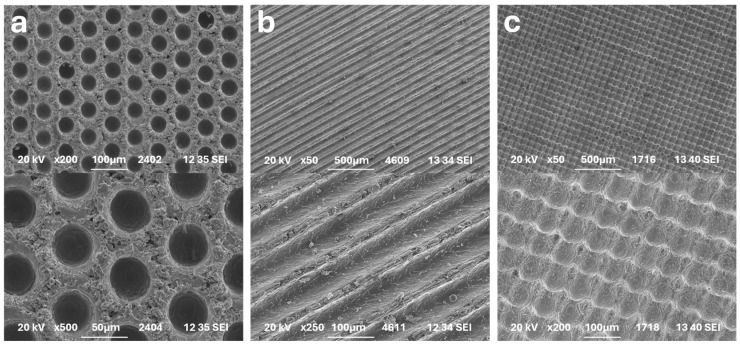
Examples of laser texturing geometries: (**a**) CSD4; (**b**) CST1; (**c**) CSC2.

Trenches geometries were produced by a 3 kW continuous laser. For laser processing, the maximal optical power of 3 kW and a scan speed of 100 m/s was used. Both structuring processes used a polygon scanner, which allows high-speed positioning of the laser beam in one direction. Polygon scanner processing is one of the key concepts for high-capacity laser machining, enabling high-precision machining at high speeds [23]. The 3 kW continuous laser with polygon scanner was also used to create the cross-trenches geometries. To create this geometry, two subsequent processes (perpendicular to each other) were needed [23]. All geometries were created in different variations of depths and widths (parameters of textures are summarized in Table 5 and Table 6 [10]). The examples of dimples, trenches and cross-trenches textures are in Figure 2.

Figure 2a shows that the dimples appear regularly spaced in a hexagonal arrangement on the surface. The trenches (Figure 2b) were formed with grooves in one direction only. This fact contributes to an increase in the speed of laser texturing. The cross-trenches texture is shown in Figure 2c. As the texture is prepared by a subsequent engraving of perpendicular lines, the structure consists of pits where both trenches meet, junctions where the material has been removed only once, and peaks that have not been worked. The rate of removing material is much higher than in the case of dimples due to the higher available laser power (see Table 5).

### 2.3. Coating Deposition Parameters

Thermal spraying of the samples was carried out at the Research and Testing Institute in Pilsen (Pilsen, Czech Republic). It was carried out using High Velocity Oxygen Fuel (HVOF) technology with a JP 5220 system (TAFA Incorporated, Torrington, CT, USA). The same previously optimized spraying parameters [26] were used for all samples. They are summarized in Table 7 [24].

### 2.4. Analysis of Laser Texturing

The analysis of laser texturing was performed using SEM (JEOL, Tokyo, Japan) and Raman spectroscopy. The microstructures were analysed on precleaned samples. The microscope used for SEM microstructures was JOEL JSM 6490LV with a voltage of 20 kV.

During laser texturing, the material may melt and possibly oxidize during removal. This oxidized ablated material can cause a number of surface imperfections. The formation of an oxide layer would be a negative factor reducing the strength of the adhesive bond between the substrate and the coating. In order to verify the presence of this oxide layer, Raman spectroscopy analysis was performed on selected samples (CSC2, NSC3 and NSD1).

The background of the instrument was also measured before the actual measurement of the samples. The Raman spectroscopic analyses were performed using a DRX microscope (located in the laboratories NTC–UWB, Pilsen, Czech Republic) by means of backscattering geometry with a green (532 nm) Nd:YVO4 DPSS laser source. The spectra were recorded in 5 different positions in the range of 50–3500 cm^−1^ with a microscope magnification of 10×, which provides a laser spot with a diameter of 2.1 µm. The laser texturing process was described in more detail in a previous paper by Kraft et al. [23].

### 2.5. Analysis of Coating–Substrate Interfaces

The quality of Stellite 6 coating–substrate interfaces was observed using optical microscopy at magnification 200× and 500× and by SEM at magnification 250×, 500× and 1000× on cross-sections prepared by standard metallographic procedures. The element distribution across the coating–substrate boundaries was analysed by EDX on two nitrided samples with cross-trenches (NSC3) and dimples (NSD1) textures. The hardness profile of pure, laser-textured and laser-textured and coated nitrided substrates was measured by HV0.3.

### 2.6. Adhesion Analysis

The adhesion of the coating was measured by tensile test according to ASTM C633-79. The test consists of the preparation of five bonded joints in which the coated specimen is glued between two rollers and placed in a special cast iron mould. HTK Ultra Bond 100 (HTK Hamburg GmbH, Hamburg, Germany) heat curable glue was used for bonding. All five samples (one set) were then cured in a heat-treatment furnace at 190 °C for 50 min. The samples were then subjected to a tensile test. The whole set of samples was subjected to visual inspection after the tensile test. The tensile strength was calculated according to the following formula, where *R_H_* stands for tensile adhesion value, *F_M_* for maximum load and *S* for the cross-section of the sample at the fracture point.

(1) $$R_{H}=\frac{F_{M}}{S}$$

## 3. Results

### 3.1. Results of Laser Texturing Analysis

**Figure 3:**
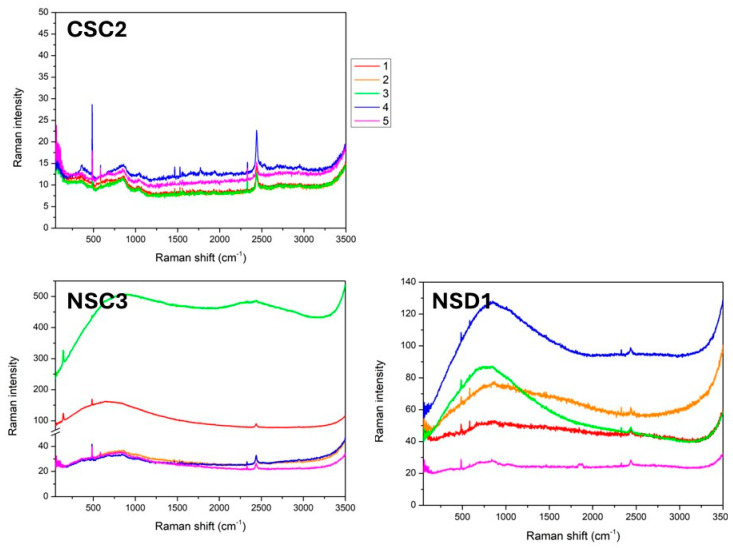
Measurement positions and measured Raman spectra on the surface of 3 types of substrates on 5 differently placed measurements.

The measurements were performed on samples CSC2, NSC3 and NSD1. The main attention was focussed on the presence of oxides and the comparison of spectra inside the laser-textured part (dimples and grids) and outside the texturing—five differently placed measurements were performed on each sample (see Figure 3).

No vibrational mode is evident in any of the measured spectra, which may be caused by either the absence of oxides or such a small presence that it leads only to a negligible vibrational response. All visible vibrational peaks are very sharp—they are caused by the influence of the background or the influence of the detector of the device. The samples on nitrided steel show a similar course of spectra compared to the sample on standard steel.

**Figure 4:**
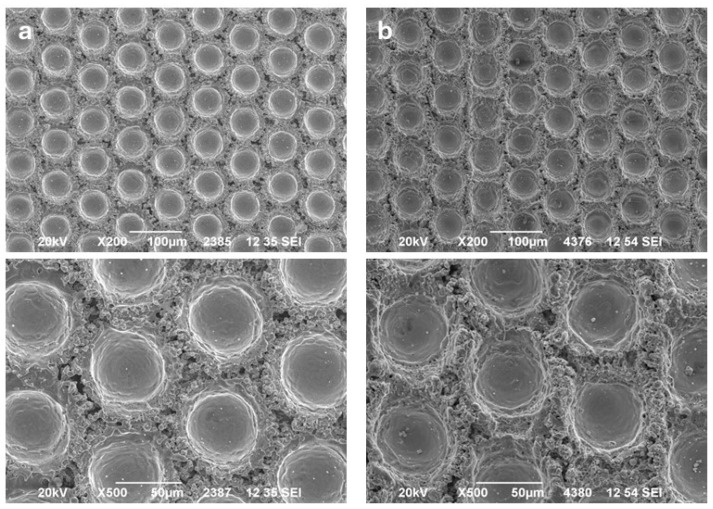
The dimple structure of (**a**) CSD1 and (**b**) NSD1.

In Figure 4, the comparison between the dimple texture made on carbon steel and nitrided steel can be seen. The dimples’ distribution, their size and mutual distances, as well as the appearance of the surrounding material seem to be similar.

### 3.2. Coating–Substrate Interface Analyses

The interfaces between deposited Stellite 6 coatings and laser-textured substrates were observed using OM (optical microscopy) on cross-sections prepared by standard metallographic procedures.

Sample photos of the microstructure of the interface of Stellite 6 coating and 1.0570 steel of each of the three types of laser texture geometry (dimples, cross-trenches and trenches) were selected for the purpose of this article.

**Figure 5:**
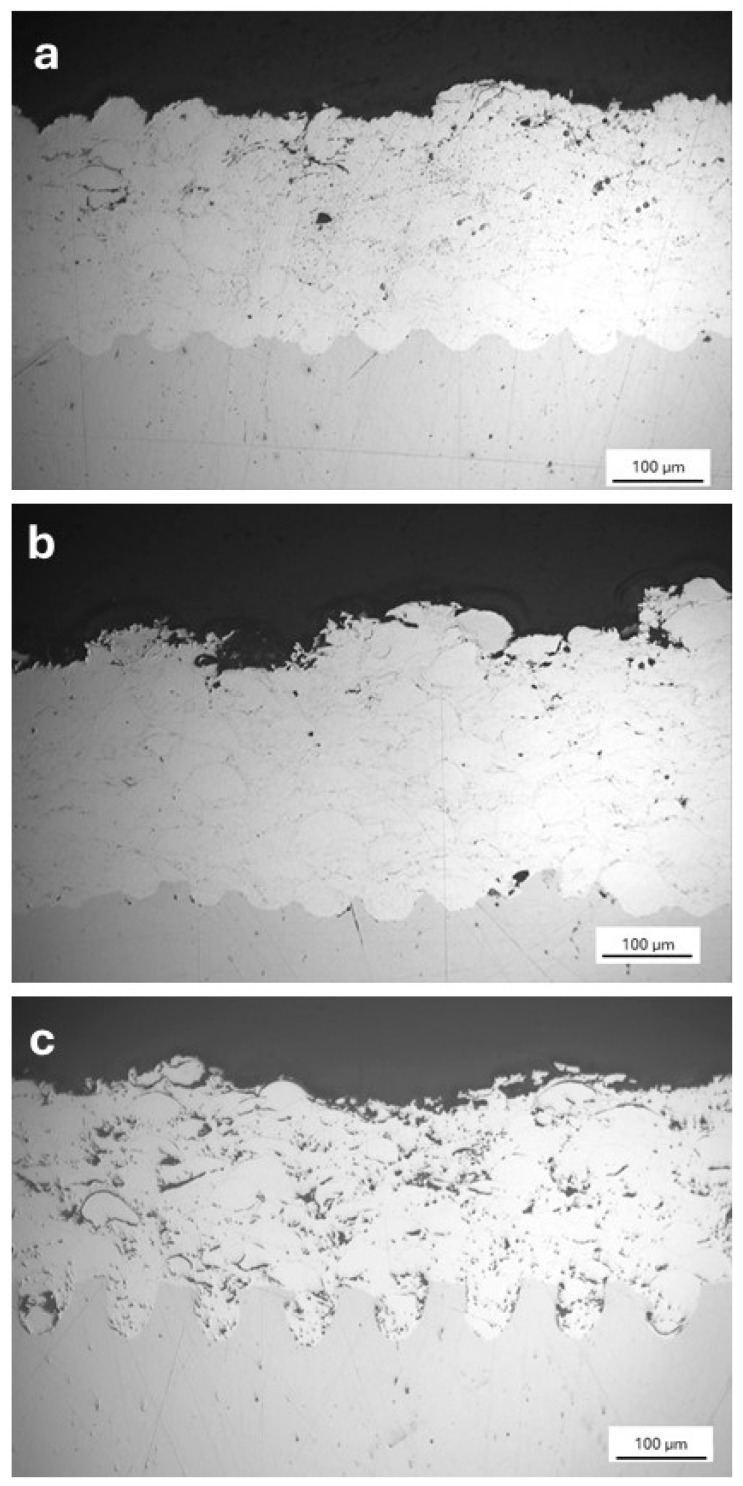
Examples of Stellite 6 coating, deposited on carbon steel substrate with various texture geometry: (**a**) CSD1, (**b**) CSC2 and (**c**) CST3.

Figure 5 shows a typical microstructure of Stellite 6 alloy deposited by HVOF technology. The coating shows characteristic semi-molten splats and visible interfacial splat boundaries. From the microstructure of sample CSD1 (Figure 5a) it can be seen that the coating has completely filled all the dimples and that there is no apparent defect or crack in it. The pore content of the coating is low, which is typical for coatings deposited by HVOF technology. A cross-section of Stellite 6 coating on sample CSC2 is shown in Figure 5b. Also, in this case, the coating completely filled the texture of the grooves, and the porosity level remained low. The filling of the grooves with coating is slightly worse in the CST3 sample than in the previous two cases. This may be due to the greater depth of the textured grooves (70 µm) than in the previous two cases (40 and 30 µm).

**Figure 6:**
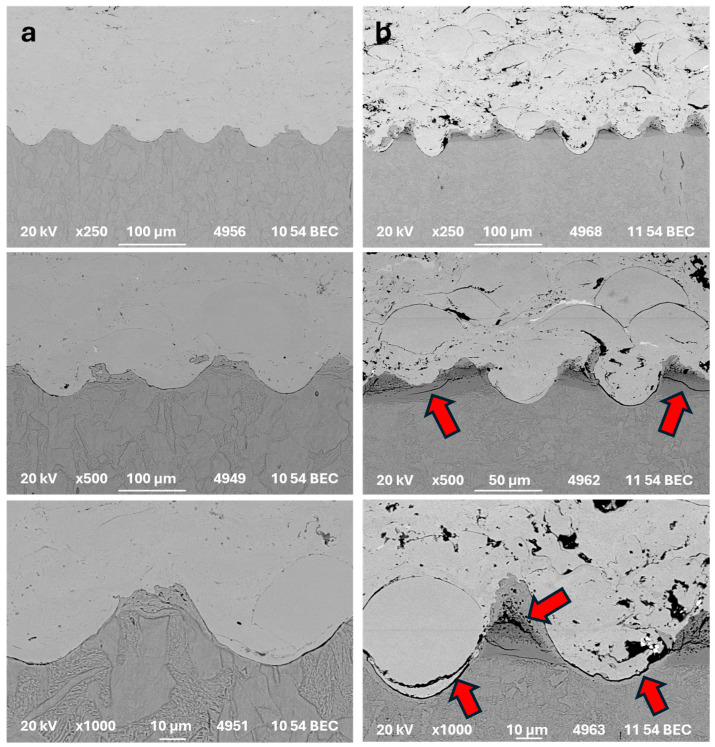
The interface between Stellite 6 coating and (**a**) carbon steel substrate CSD1 and (**b**) nitride steel NSD1. The red arrows indicate multiple cracks in the thin layer beneath the surface of the nitrided substrate.

In Figure 6, the SEM of the interface between the Stellite 6 coating and the carbon steel and nitrided steel, both with the same texture (Dimple 1), is presented.

Even though the laser parameters were set to produce a similar surface texture on both substrates, and the surface analyses confirm the similarity (see Figure 5), the interfaces between the deposited coating and substrates differ.

For carbon steel substrate, the coating adhered to the substrate without cracks or decohesion. The regularity of the quality is high, and there is no defect observed in the underlaying material.

On the contrary, in the case of nitrided substrate, the depths of the dimples differ. Moreover, many cracks appeared in the thin layer under the surface. This layer interacted differently with the laser beam, resulting in dimples with lower depths. Also, a number of subsurface cracks can be seen. Except for the scars in the substrate material, the decohesion between the coating particles and substrate is also detectable.

**Figure 7:**
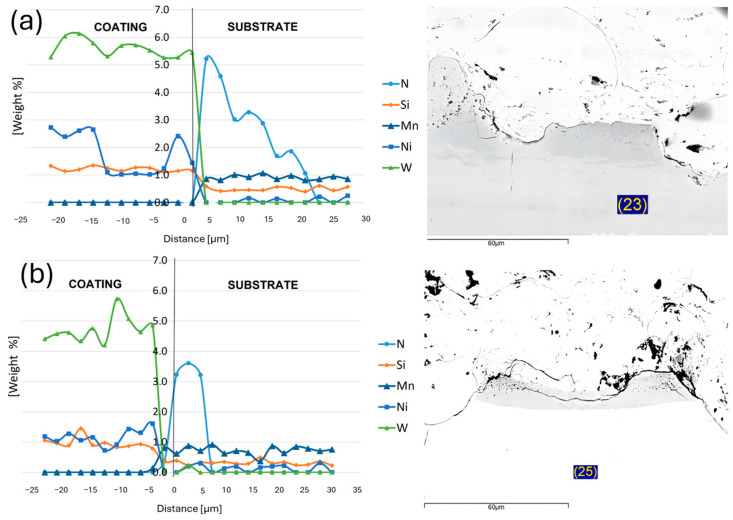
Graph of the EDX analysis on (**a**) NSD1 and (**b**) NSC3. The numbers 23 and 25 in the figure represent the total number of measurements taken.

To analyze the interface in more detail, the EDX analysis was performed on two nitrided samples with different laser textures—NSD1 and NSC3. A total of 25 and 23 measurements were taken on both the samples, respectively. The results can be seen in Figure 7.

The main reason for this analysis was to determine if there was any diffusion between coating and substrate elements. The measurement showed that there is no diffusion in the content of typical elements (W for coatings and Ni or Mn for substrate); neither the N diffused to the counter material. The increase in nitrogen occurred up to the substrate area. The thin subsurface layer, containing an increased amount of N, varied between 5 and 20 µm.

**Figure 8:**
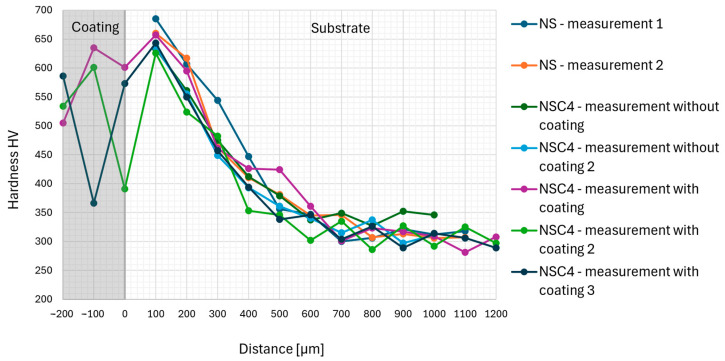
HV0.3 distribution for nitride (NS—2 measurements), laser-textured (NSC4 without coating—2 measurements), and laser-textured and sprayed samples (NSC4 with coating—3 measurements).

Except for element distribution across the coating–substrate cross section, the hardness evolution was measured on the nitride substrates without any treatment (NS), laser-textured (NSC4 without coating) and laser-textured and sprayed (NSC4 with coating). Two measurements were taken at different locations for both the untreated nitrided substrate and the laser-textured substrate. For the laser-textured, coated substrate, three measurements were taken at three different locations. The HV distribution can be seen in Figure 8. The hardness of all measured samples was the highest close to the surface. The thickness of the hardened layer reached similar values for all tested samples. The hardness of the Stellite 6 coating was lower compared to the hardened layer but higher than the core material. The scatter of the HV0.3 value within the coating is caused by the heterogeneous character of the coating. The hardness very close to the interface was lowered by laser texturing, compared to non-textured samples. This is caused by breaking the homogeneity of the thin ultra-hard layer with a high content of nitrogen.

### 3.3. Adhesion Analysis Results

The effect of laser texturing on the resulting adhesion of Stellite 6 (Amperit 2637-02) was investigated using mechanical testing and surface analysis. In this experiment, samples with different laser texturing parameters were compared with samples treated conventionally. The possibility of laser texturing of nitrided samples was also investigated. Table 8 and Table 9 give an overview of the laser-textured samples with different geometries and dimensions and their adhesion in MPa. In addition, a set of grit-blasted samples is included for comparison.

## 4. Discussion

The results of the adhesion tensile tests showed that the adhesive strength of laser-textured carbon steel substrates is sufficient, similar to grit-blasted substrates, while the nitrided steel substrate has lower adhesive strength, no matter if it is textured or grit blasted.

Table 8 shows the adhesion results of Stellite 6 coating on carbon steel samples. It is evident that the highest adhesion values were achieved for the CSD1 (72.8 MPa) samples. Other sets of samples that achieved higher adhesion values are CSD4 (64.7 MPa), CSC2 (66.1 MPa) and CST3 (65.2 MPa). Almost all of these samples had a fracture in the glue during the adhesion tensile test. From this result it can only be concluded that the adhesion of the coating is higher than the adhesion of the glue. On the contrary, low adhesion values were measured for the CSD3 (45.1 MPa) and CSC1 (34 MPa) samples. This is probably due to inappropriately chosen parameters of the textures. In the case of samples CSD3, the depth of the dimples was probably too large (100 µm), which may have caused insufficient attachment of the powder particles during thermal spraying. An inappropriate combination of the diameter and depth of the dimples could also be a problem. For the CSC1 samples, the problem is probably a too small depth of the engraved texture (15 µm).

Table 9 shows the adhesion results of the Stellite 6 coating on nitrided samples. Even with the same texture parameters, lower adhesion values were obtained for the nitrided samples (for example, sample CSD1—72.8 MPa; sample NSD1—40.1 MPa).

Also, grit-blasted carbon steel samples were compared with grit-blasted nitrided samples. Again, lower adhesion values were measured for nitrided samples. It is clear from the results obtained that both texturing and nitriding parameters have a great influence on the resulting adhesion of the coating.

In general, it can be summarized that the best average adhesion results were achieved with structures created with slow laser texturing speeds. Kromer et al. [27] investigated the adhesion of Ni-Al coating on aluminum alloy substrate produced by atmospheric plasma spraying. Surface topographies were obtained by grit blasting and laser texturing. The results showed that laser texturing increased the adhesive bond strength. The available literature mentioned above shows that higher adhesion values can be obtained with an optimized laser texturing process than with conventional surface treatments. The resulting adhesion value, however, is highly dependent on the laser texture parameters, especially the diameter and depth of the textured geometry.

The possible negative influence of potential oxidation of the textured surface on the adhesive strength has to be taken into consideration. The possible formation of unwanted oxides is a discussed problem in the case of laser texturing. Costil et al. [28] investigated the effect of laser beam interaction on an aluminum alloy surface. A more detailed surface analysis was carried out by XPS (X-ray photoelectron spectroscopy) measurements. Their results showed that the parameters with the greatest influence on the morphology of the surface are laser power and laser resolution. The other parameters such as laser frequency and scanning velocity demonstrated a lower impact on the surface. Moura et al. [29] analysed the characteristics of a laser-textured surface of Ti-6Al-4V. They used X-ray diffraction to detect the presence of oxide layers. The results showed the formation of α-Ti, Ti_6_O and TiO_2_ phases on the textured surface. In the case of samples analysed in this study, the presence of unwanted oxides was excluded by Raman spectroscopy analysis.

EDX analysis of the nitrided samples was performed to determine if there was any diffusion of nitrides from the substrate into the coating, which could negatively affect the properties of the coating. No information on thermal spraying of nitrided samples was found in the available literature. This was also one of the reasons for performing the EDX analysis. The temperature of the nitriding process was in the range of 495–565 °C for all types of steel [30]. The samples were not heated to such temperatures during thermal spraying. Therefore, the conditions for diffusion of nitrides from the substrate into the coating were not met. The graphs in Figure 7 show that the amount of nitrogen increased only in the substrate area. Thus, it can be inferred that no diffusion of nitrides into the coating occurred for those samples. From the measured adhesion results, it is clear that even within the nitrided samples there is a considerable dispersion of values.

The main reason for the lower adhesion of the coating on the nitrided substrate is the presence of a thin, hard nitrided layer, which is subject to breakage both during laser texturing and probably also during grit blasting. The reason for the lower adhesion is not necessarily the lower achieved surface roughness but the existence of brittle failures in the subsurface nitrided coating, as can be seen in Figure 6.

## 5. Conclusions

The findings of this study can be summarized as follows:

- Geometry and texturing parameters have a huge influence on the resulting adhesion of the coating. The use of 200 W ns laser for texturing dimples produced better adhesion results than texturing cross-trenches with a 3 kW continuous laser with polygon scanner, but the speed process of texturing dimples is significantly slower. The solution could be to optimize the texture parameters of the trenches, for which promising adhesion results have been obtained and the process speed is close to the conventional preparation method.
- One of the most discussed problems of laser texturing is the formation of unwanted oxides. Oxidation of the ablated material and associated problems do not always have to occur after the laser texturing. It depends mainly on the texturing parameters such as laser power and laser resolution. Oxides were not found in our case (for dimple structure).
- The nitriding treatment of the samples before thermal spraying negatively affected the resulting adhesion of the coating. In all cases the adhesion was significantly lower than in the case of non-nitrided samples, even with the same texturing parameters. The probable reason for lower adhesion is an occurrence of cracks in the nitride layer.
- During the thermal spraying of nitrided samples, there is no diffusion of nitrides between the substrate and the coating. The reason is the low temperature of the samples during thermal spraying, which does not meet the conditions for diffusion.

## Figures and Tables

**Table 1:** Chemical composition of low carbon steel 1.0570 and nitrided steel 42CrMo4 [24,25].

Chemical Composition [%]	C	Mn	Si	P	S	Cr	Mo
1.0570	Max. 0.2	Max. 1.6	Max. 0.55	Max. 0.04	Max. 0.045	0	0
42CrMo4	0.38–0.45	0.6–0.9	Max. 0.4	Max. 0.025	Max. 0.035	0.9–1.2	0.15–0.3

**Table 2:** Mechanical properties of low carbon steel 1.0570 and nitrided steel 42CrMo4 [24,25].

Mechanical Properties	Tensile Strength	Yield Strength	Hardness	Tensibility
1.0570	Max. 630 MPa	Max. 345 MPa	Max. 190 HB	Max. 22%
42CrMo4	Max. 1300 MPa	Max. 800 MPa	Max. 217 HB	Max. 15%

**Table 3:** Overview of grit-blasted samples.

Designation	Surface Pre-Treatment	Substrate	Heat Treatment	Grit Blasting Medium
CSG	Grit blasted	1.0570	Nitrided	Corundum (F22)
NSG	Grit blasted	42CrMo4	Nitrided	Corundum (F22)
NSG	Grit blasted 3	42CrMo4		Corundum (F22)

**Table 4:** Nominal parameters of laser sources.

Model/Parameters	YLR-3000-SM	YLP-HP
Temporal domain	Continuous wave	Nanosecond
Manufacture	IPG	IPG
Pulse duration	-	30–240 ns
Wavelength	1070 nm	1070 nm
Max. optical power	3000 W	200 W
Max. rep. frequency	-	1 MHz

**Table 5:** Laser texture parameters used on 1.0570 steel substrate.

Designation	Texture	Width [µm]	Depth [µm]	Spacing [µm]	Surface Proc. Rate [mm^2^/s]
CSD1	Dimple 1	80	40	90	74
CSD2	Dimple 2	80	70	85	29
CSD3	Dimple 3	80	100	80	18
CSD4	Dimple 4	70	70	70	34
CSD5	Dimple 5	100	70	100	47
CSC1	Cross-trenches 1	70	15	80	325
CSC2	Cross-trenches 2	70	30	75	163
CST1	Trenches 1	70	50	80	247
CST2	Trenches 2	70	50	100	307
CST3	Trenches 3	70	70	100	213

**Table 6:** Laser texture parameters used on 42CrMo4 (nitrided) substrate.

Designation	Texture	Width [µm]	Depth [µm]	Spacing [µm]	Surface Proc. Rate [mm^2^/s]
NSD1	Dimple 1	80	40	90	74
NSC3	Cross-trenches 3	70	50	80	108
NSC4	Cross-trenches 4	70	75	70	107

**Table 7:** Thermal spray parameters of Amperit 2637-02 powder.

Deposition Parameters	
Oxygen	996 L/min
Kerosene	27 L/h
Deposition distance	360 mm
Barrel	6″
Amount of powder	45 ± 5 g/min
Carrier gas	Nitrogen
Carrier gas flow	5 L/min
Surface speed	33 m/min
Offset	6 mm
Combustion chamber pressure	8 bar

**Table 8:** Adhesion tensile test results of Stellite 6 coating on 1.0570 steel substrate.

Designation	Texture	Ø Adhesion [MPa]	Fracture Type
CSD1	Dimple 1	72.8 ± 9.2	In glue
CSD2	Dimple 2	60.2 ± 10.3	In glue
CSD3	Dimple 3	45.1 ± 5.1	In glue
CSD4	Dimple 4	64.7 ± 7.8	In glue
CSD5	Dimple 5	54 ± 11.7	In glue
CSC1	Cross-trenches 1	34 ± 8.1	Cohesive
CSC2	Cross-trenches 2	66.1 ± 16.9	In glue, cohesive–adhesive
CST1	Trenches 1	54.4 ± 8.9	In glue
CST2	Trenches 2	49.8 ± 13.2	In glue
CST3	Trenches 3	65.2 ± 7.6	In glue
CSG	Grit blasted 3	63.4 ± 5.2	In glue

**Table 9:** Adhesion tensile test results of Stellite 6 coating on 42CrMo4 (nitrided) steel substrate.

Designation	Texture	Ø Adhesion [MPa]	Fracture Type
NSC3	Cross-trenches 3	52.4 ± 6.7	In glue, cohesive–adhesive
NSD1	Dimple 6	40.1 ± 2.7	In glue, cohesive–adhesive
NSC4	Cross-trenches 4	34.8 ± 11.4	In glue, cohesive–adhesive
NSG1	Grit blasted 1	35.6 ± 6.1	Adhesive
NSG2	Grit blasted 2	48.7 ± 11.3	Adhesive

## Data Availability

The original contributions presented in the study are included in the article, further inquiries can be directed to the corresponding author.
